# Case Report and Literature review. Neuropsychiatry Manifestation of Frontal Lobe Neoplasm- Meningioma. Prevalence, Presentation and Pathogenesis

**DOI:** 10.1192/j.eurpsy.2024.1340

**Published:** 2024-08-27

**Authors:** U. Anyeji, O. Alli-Balogun

**Affiliations:** Psychiatry, BronxCare Health System, Bronx, United States

## Abstract

**Introduction:**

Meningiomas are the most frequent primary brain tumor. Although most Meningiomas are benign, their location in the central nervous system can predict symptomatology which could result in significant morbidity and mortality. However, due to the slow-growing nature, meningiomas are usually asymptomatic, and diagnosis is often made incidentally on neuroimaging or at an autopsy. The incidence rate is 1.2-fold higher in Black Americans than White Americans. Neuropsychiatry manifestation might be only initial presentation; thus, psychiatrists are often the first to see these patients, and the correct diagnosis may be made only when the tumor has grown to a considerable size and begun to displace the brain.

**Objectives:**

The aim of this study is to understand the biological basis of psychiatry symptoms in patients with Frontal Lobe meningiomas.

**Methods:**

A review of literature and individual patient data analysis was conducted. The literature review was conducted on PubMed, Medline, MeSH, Google Scholar, and mount Sinai’s levy Library using the key words; meningioma, meningioma with psychiatric symptoms, psychosis, depression, neuropsychiatry manifestation of meningiomas.

**Results:**

The review revealed that 88% of brain tumors and psychiatric symptoms are located in the frontal region. Meningiomas accounts for 13%-26% of intracranial tumors. There is a reported low incidence due to its slow growing nature and are usually asymptomatic. Incidence of meningiomas is predominant in females, and is attributed to hormonal factors, this is associated with estrogen and progestogen cycles. Reports shows that smoking has been linked to increase risk of meningiomas in men. Frontal lobe meningiomas may present with only psychological symptoms that resemble depression, anxiety states, hypomania and schizophrenia. Personality and mental status changes are also noted in Frontal lobe tumors. Left sided lesions are associated with inhibition of motor activity, impairment in motor and initiative aspect of speech, diminished generalization ability and general inertia of mental process.

**Conclusions:**

Given the absence of frank neurological symptoms, to help localize the lesion, most meningiomas are missed due to diagnostic overshadowing of the primary psychiatric illness. Peritumoral edema indicates the underlying mechanism and location of the lesion predicts symptomatology. Like our patient who is an 81-year-old male with no past psychiatry history, presenting to our comprehensive psychiatry emergency program with psychiatric manifestation as the initial presentation and subsequently with MRI suggestive of Right Frontal extra-axial meningioma. This study shows that primitive frontal lobe tumors are likely to be misdiagnosed as patients with such tumors are often referred first to psychiatrist. High index of suspicious is needed.

**Disclosure of Interest:**

None Declared

